# Gadoxetic Acid-Enhanced MRI and Sonoelastography: Non-Invasive Assessments of Chemoprevention of Liver Fibrosis in Thioacetamide-Induced Rats with Sho-Saiko-To

**DOI:** 10.1371/journal.pone.0114756

**Published:** 2014-12-09

**Authors:** Ya-Wen Chen, Meng-Yuan Tsai, Huay-Ben Pan, Hui-Hwa Tseng, Yu-Ting Hung, Chen-Pin Chou

**Affiliations:** 1 National Institute of Cancer Research, National Health Research Institutes, Miaoli, 350, Taiwan; 2 Graduate Institute of Basic Medical Science, China Medical University, Taichung, 404, Taiwan; 3 Department of Radiology, Kaohsiung Veterans General Hospital, Kaohsiung, 813, Taiwan; 4 School of Medicine, National Yang-Ming University, Taipei, 112, Taiwan; 5 Department of Medical Imaging and Radiological Sciences, I-Shou University, Kaohsiung, 824, Taiwan; 6 Department of Pathology, Kaohsiung Veterans General Hospital, Kaohsiung, 813, Taiwan; 7 Department of Medical Laboratory Sciences and Biotechnology, Fooyin University, Kaohsiung, 807, Taiwan; University of Modena & Reggio Emilia, Italy

## Abstract

**Background:**

This study aimed to compare the performance of gadoxetic acid -enhanced **magnetic resonance imaging** (MRI) and sonoelastography in evaluating chemopreventive effects of Sho-Saiko-To (SST) in thioacetamide (TAA)-induced early liver fibrosis in rats.

**Materials and Methods:**

Ten of Sprague-Dawley rats receiving TAA (200 mg/kg of body weight) intraperitoneal injection were divided into three groups: Group 1 (TAA only, n = 3), Group 2 (TAA +0.25 g/kg SST, n = 4) and Group 3 (TAA+1 g/kg SST, n = 3). Core needle liver biopsy at week 2 and liver specimens after sacrifice at week 6 confirmed liver fibrosis using histological examinations, including Sirius red staining, Ishak and Metavir scoring systems. Gadoxetic acid-enhanced MRI and shear-wave sonoelastography were employed to evaluate liver fibrosis. The expression of hepatic transporter organic anion transporter 1 (Oatp1), multidrug-resistant protein 2 (Mrp2) and alpha-smooth muscle actin (α-Sma) were also analyzed in each group by immunohistochemistry (IHC) and Western blot.

**Results:**

According to histological grading by Sirius red staining, Ishak scores of liver fibrosis in Groups 1, 2 and 3 were 3, 2 and 1, respectively. As shown in gadoxetic acid-enhanced MRI, the ratio of relative enhancement was significantly lower in Group 1 (1.87±0.21) than in Group 2 of low-dose (2.82±0.25) and Group 3 of high-dose (2.72±0.12) SST treatment at 10 minutes after gadoxetic acid intravenous injection (p<0.05). Sonoelastography showed that the mean difference before and after experiments in Groups 1, 2 and 3 were 4.66±0.1, 4.4±0.57 and 3±0.4 KPa (p<0.1), respectively. Chemopreventive effects of SST reduced the Mrp2 protein level (p<0.01) but not Oatp1 and α-Sma levels.

**Conclusion:**

Sonoelastography and gadoxetic acid-enhanced MRI could monitor the treatment effect of SST in an animal model of early hepatic fibrosis.

## Introduction

Cirrhosis of liver is a common end consequence of a variety of chronic liver diseases. Its underlying pathology, fibrosis, represents the common response of liver to toxic, infectious, or metabolic agents. Hepatic fibrosis is the excessive accumulation of extracellular matrix proteins including collagen that occurs in most types of chronic liver diseases. Advanced liver fibrosis results in cirrhosis, hepatic failure, and portal hypertension and often requires liver transplantation. Numerous antifibrotic agents that may delay progression to decompensate cirrhosis or even reverse cirrhosis are currently developed; however, progress has been impeded by the small number of tools available for measuring efficiently the progression or reversal of fibrosis [1].

Thioacetamide (TAA) is a model of liver cirrhosis that induces a number of metabolic and histological alterations similar to human diseases [2], [3]. Chemoprevention of experimental TAA-induced rat liver damage using dietary supplementation or cyclooxygenase-2 inhibitor was proposed [2],[3]. Sho-Saiko-To (SST, also known as Minor Bupleurum Formula and Xiao Chai Hu Tang in Chinese), a traditional commonly used Chinese herbal medicine mixture, has long been used by patients with chronic liver diseases, such as chronic hepatitis and cirrhosis, in Asia [4]. Sho-Saiko-To can prevent liver damage and liver fibrosis through anti-inflammation and anti-oxidant. Moreover, it can promote liver regeneration [4], and has been known to improve immune system and prevent cancer development and metastases [5], [6]. A clinical human trial (NCT00590564) of SST was proposed to evaluate liver function and viral load in patients with chronic hepatitis C [7].

Staging of liver fibrosis is crucial in the management of patients with chronic liver diseases since severity of fibrosis influences the prognosis and treatment options. An early diagnosis of cirrhosis is particularly important in patients with compensated chronic liver diseases, because it triggers screening for portal hypertension and hepatocellular carcinoma. Invasive biopsy is thus considered the gold standard for diagnosing and staging cirrhosis. The risk of disease progression can be monitored by the sequential histological grading of inflammation as well as by the staging of fibrosis [1]. However, liver biopsy is poorly accepted by patients and has a risk of various complications, such as hemorrhage and infection. In addition, liver biopsies may be prone to sampling errors and inter-observer variability due to subjective morphological evaluation in limited specimen [8]. Therefore, non-invasive quantitative markers or diagnostic tests are required to assess the presence and severity of liver cirrhosis. Ideally, a non-invasive marker of liver fibrosis should be liver-specific, easy to perform, reliable and inexpensive. In addition, it should be accurate not only for the grading of fibrosis, but also for monitoring disease progression and treatment efficacy. Several non-invasive diagnostic methods for fibrosis or cirrhosis, including clinical signs, sonographic signs, or biochemical blood parameters, have been evaluated. The latest technological advance in this setting is the measurement of uptake of contrast agents using MRI and liver stiffness by means of transient sonoelastography.

Gadolinium ethoxybenzyl diethylenetriaminepentaacetic acid (Gd-EOB-DTPA, *Primovist*, gadoxetic acid) is a hepatocyte-specific contrast agent for MRI of the liver. The additional value of Gd-EOB-DTPA compared with other gadolinium-based contrast agents is the selective uptake of the contrast medium by functioning hepatocytes. In turn, this uptake can be employed to acquire MR images during the hepatobiliary phase, which occurs approximately 15 to 20 min after the injection of the contrast medium [9]. Therefore, this agent can be used for both dynamic bolus-phase MRI and liver-specific (hepatocyte-phase) MRI in the same examination performed within a reasonable time frame. In other words, gadoxetic acid combines the properties of an extracellular and a hepatocyte-specific contrast agent [10]. Sonoelastography which is integrated into a conventional ultrasound platform has recently been applied as a new and promising non-invasive tool for measuring liver stiffness and as an accurate marker for predicting the degree of liver fibrosis [11], [12]. Additionally, the utility of elastography in monitoring progression of fibrosis has also been evaluated [13].

Currently, only limited imaging tools can be employed to assess the efficacy of developing antifibrotic medication and the necessity of health care. Investigating the diagnostic performance of liver fibrosis using sonoelastography and gadoxetic acid-MRI is the key driving forces. In this study, early stages (Ishak scores 1–3/Metavir scores F1–F2) of fibrotic livers were developed by administration of SST in rats with TAA-induced liver fibrosis in order to evaluate non-invasive imaging modalities, including sonoelastography and gadoxetic acid-enhanced MRI for assessments of early liver fibrosis.

## Materials and Methods

### SST reagents

The SST compound (No. 45353615), purchased from Kaiser Pharmaceutical Co. (Tainan, Taiwan), is a mixture prepared with the following seven herbal medicines: 5.6 g of the root of *Bupleurum falcutum*, 5.6 g of the tuber of *Pinellia teammate*, 2.1 g of the root of *Scutellaria baicalensis*, 2.1 g of the fruit of *Zizyphus jujube*, 2.1 g of the root of Panax ginseng, 2.1 g of the root of Glycyrrhiza and 2.1 g of the rhizome of *Zingiber officinale* [14].

### Ethics statement and animals

This animal study was performed in strict accordance with the recommendations in the guidelines for the Care and Use of Laboratory Animals of Kaohsiung Veterans General Hospital, Taiwan. The protocol was approved by the Institutional Animal Care and Use Committee (IACUC) of Kaohsiung Veterans General Hospital (Protocol No: vghks-101-A015). All animals were housed with enough food and water. All surgery was performed under isoflurane anesthesia, and sacrifice was conducted under carbon dioxide euthanasia. All efforts were made to minimize suffering in accordance with the ARRIVE (Animal Research: Reporting of *In Vivo* Experiments) guidelines.

**Figure 1:**
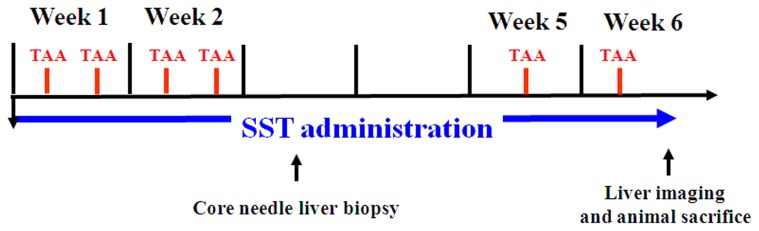
Experimental protocols of thioacetamide (TAA)-induced hepatic fibrosis in rats pretreated with Sho-Saiko-To (SST). Eight-week-old male rats were intraperitoneally injected with TAA (200 mg/kg) twice weekly at weeks 1 and 2 and then once at weeks 5 and 6. A cohort of rats receiving vehicle without treatment of SST (TAA only) was assigned as Group 1. Group 2 (TAA +0.25 g/kg SST) was administered moderate dose (0.25 g/kg) of SST two days before the first TAA exposure and sustained for 6 weeks. Group 3 (TAA +1 g/kg SST) was administered high dose (1 g/kg) of SST.

Eight-week-old inbred male Sprague-Dawley (SD) rats weighting approximately 250–300 g were purchased from the National Animal Center (Taipei, Taiwan) and kept on a standard rat diet with free access to tap water and food, with a 12-h light-dark cycle. An experiment was designed as a 6-week protocol to examine the chemoprevention effect of SST on early pathologic stages of liver fibrosis. To avoid the acute effect of thioacetamide (TAA, Sigma, St Louis, MO, USA)-induced liver injury rats [15], a modified TAA-induced liver fibrosis was performed to mimic chronic liver fibrosis. Chronic liver damage was induced by intermittent intraperitoneal injection of **TAA** (200 mg/kg of body weight) during 6 weeks (Fig. 1). The **intermittent intraperitoneal** injection causes less acute illness in rats.

This experiment was conducted on three groups: Group 1 (n = 3) received TAA only, Group 2 (n = 4) received TAA and 0.25 g/kg SST, and Group 3 (n = 3) received TAA and 1 g/kg SST in drinking water. Another three SD male rats purchased at the same time were used as normal controls for blood test and histological analyses. Early liver fibrosis was confirmed by ultrasound-guided biopsy in one rat of each group at week 2. The core liver biopsy was performed under general anesthesia with an 18 G semi-automatic biopsy Tru-cut system. Rats were sacrificed at week 6 and the liver samples were immediately frozen stored at −80°C. For immunohistochemical examinations, liver samples were fixed in 10% neutral buffered formalin, processed through a series of graded alcohols, and embedded in paraffin. Serum samples were frozen in liquid nitrogen and stored at −80°C.

### Liver histology and quantification of liver fibrosis

Liver biopsy specimens were fixed in formalin and embedded in paraffin. For histological examination, the sections (5 µm thick) were cut and collected on polylysine-coated slides. The dried sections were de-waxed in xylene, rehydrated in an alcohol series of a decreasing concentration and stained with hematoxylin/eosin (H&E, Sigma). To measure the hepatic collagen content in liver fibrosis, all deparaffinized liver slices were incubated in 0.1% Fast Green FCF (in saturated picric acid, Sigma) for 15 minutes, and then incubated in 0.1% Sirius red (in saturated picric acid, Sigma) for 40 minutes. Images of liver sections in each group were photographed at 1,024×768 pixels with 0.23 µm×0.23 µm of pixel size using the Olympus BX60 microscope and digital camera system (DP-70, Olympus, Tokyo, Japan). All images were analyzed using the ImageJ software (version 1.45b; National Institutes of Health, Bethesda, MD, USA) and the standard threshold algorithm. The whole area and the fibrosis area stained with Sirius red were measured. The percentage (%) of intensity in Sirius red staining was calculated with the following formula: fibrosis area/whole area×100.

Liver fibrosis was categorized semi-quantitatively following both Ishak and Metavir scoring systems. According to the Ishak staging system, fibrosis was scored on a 0–6 scale as follows: 0: no fibrosis, 1: fibrous expansion of some portal areas, with or without short fibrous septa; 2: fibrous expansion of most portal areas, with or without short fibrous septa; 3: fibrous expansion of most portal areas with occasional portal to portal (P-P) bridging; 4: fibrous expansion of portal areas with marked bridging portal to portal (P-P) as well as portal to central (P-C); 5: marked bridging (P-P and/or P-C) with occasional nodules (incomplete cirrhosis); and 6: cirrhosis [16]. In accordance with the Metavir scoring system, fibrosis was staged on a 0–4 scale as follows: F0: no fibrosis; F1: portal fibrosis without portal septa; F2: portal fibrosis with few septa; F3: numerous septa with- out cirrhosis; and F4: cirrhosis [17].

### Immunohistochemistry (IHC)

To visualize antigen, the de-waxed sections were heated in a microwave for 20 min in a citrate buffer (pH 6.0) and incubated with 3% hydrogen peroxide to block the endogenous activity. The samples were reacted with monoclonal antibodies: anti-Mrp2 (Abcam, Cambridge, MA, USA), anti-Oatp1 (Alpha Diagnostic International, San Antonio, TX, USA) and anti-α-Sma (Abcam). Horseradish peroxidase/Fab polymer conjugate (Polymer detection system, Zymed Lab, Invitrogen, Carlsbad, CA, USA) was then applied to the sections and the sections were incubated for 10 min. After rinsing with PBS, the sections were incubated with peroxidase substrate diaminobenzidine (1∶20 dilution, Zymed Lab) for 5 min and counterstained with hematoxylin for 2 seconds, dehydrated with serial ethyl alcohol, cleared with xylene, and finally mounted. All illustrations were composed using Adobe Photoshop software (version 6.0, Adobe Systems, Mountain View, CA, USA), adjusting only brightness and contrast for optimal visualization. The intensity of positive signals was quantified as described previously. The percentage (%) of intensity in positive signals was calculated with the following formula: positive area/whole area×100.

### Gadoxetic acid-enhanced MRI

After 6 weeks of oral administration of SST, each rat was imaged on a 1.5-T clinical MRI scanner (General Electric Medical Systems, Milwaukee, WI, USA) and an 8-channel phased-array wrist coil (General Electric). Before MRI scanning, rats were anesthetized, catheterized and center of the MRI gantry in a wrist coil. The axial images of the whole rat liver were obtained using a fat-saturated FSE T2-weighted sequence (TR/TE 6000/99; matrix 128×128; field of view [FOV] 80×80 mm; NEX = 8; echo train length = 32; ASSET = 2). Gadoxetic acid-enhanced dynamic liver MRI with T1-weighted spoiled gradient recalled acquisition in steady state (SPGR) was performed before and after intravenous contrast agent venous injection. Each rat was given an intravenous bolus injection of gadoxetic acid (*Primovist*, Bayer Schering Pharma AG, Berlin, Germany) as contrast agent at a dose of 25 µmol/kg of body weight at a flow rate of 0.5 mL/s manually, followed by a 0.5-mL saline flush via the tail vein. Dynamic liver MRI acquisitions were performed with the following parameters: FOV = 160 mm×80 mm, matrix = 256×128, TR/TE = 8/5 and slice thickness = 3 mm. Seven sequential liver images including one pre-contrast images and six post-contrast images were taken at 10, 20, 30, 40, 50, 60 min after injection of gadoxetic acid.

Signal intensity (SI) was measured on a pixel-by-pixel basis using commercially available FuncTool software (**Advanced Workstation** 4.2, **GE**). Two experienced radiologists with **more than** 10 **years** of **experience in liver imaging** measured the signal intensity of the rat liver by consensus at five different regions of interest (**ROI**) of the hepatic parenchyma, **avoiding enhancement of vessels** and bile structures. The mean SI of each ROI was recorded for analysis. The relative enhancement (RE) ratio was calculated with the following equation: RE = [(SIpost-background)/(SIpre-background)]×100, where SIpre and SIpost were signal intensities of the liver before and after injection of the contrast agent. The final results of mean RE of the five ROIs were **expressed** as **mean** ± standard deviations. The maximum RE (SImax) in the rat liver was determined during signal intensity measurement. The T1 signal drop was expressed as the percentage values of gadoxetic acid enhancement reduction in liver from maximum signal intensity (speed of excretion through the bile ducts) in each late dynamic phase and was calculated as [(SImax-SIpost)/(SImax)]×100.

### Sonoelastography

Before and after 6 weeks of oral administration of SST, each rat was imaged using a sonoelastography scanner. Sonoelastography of liver was performed on anaesthetized but spontaneously breathing rats (4% isoflurane in air maintained at 2%). Sonoelastography was performed using a freehand technique at the same time as gray-scale sonography performed by the same radiologist. Liver stiffness measurements were performed using the Aixplorer sonography system (SuperSonic Imagine, Aix en Provence, France) and its ShearWave elastography (SWE) mode. Sonoelastography was employed to evaluate a real-time tissue stiffness of liver parenchyma. The probe used for the B-mode grayscale and shear-wave sonoelastography had a frequency range of 15 MHz. The combined conventional gray-scale liver imaging and sonoelastographic examination took about 10–15 minutes. The probe was placed very lightly by applying a large amount of sonography jelly and avoiding artifacts of stiffness radiating from the contact area and hand motion. For liver stiffness measurement, the probe was kept still at the selected area for 5 to 10 seconds during sonoelastographic scanning. A dynamic color-coded evaluation of liver stiffness and mean stiffness of region of interests (ROIs) were measured in kilopascal (KPa) by consensus of two radiologists who had more than 10 years of experience in performing liver sonography. Totally, five circular ROIs measured 0.5 cm in diameter on sonoelastographic images were obtained from liver parenchyma. All ROIs were drawn and the proper measurement areas were identified. Before TAA and SST medication, a baseline sonoelastography was made. At the end of the experiment, a final sonoelastography was performed on the same day when the rat was sacrificed. The difference in mean elasticity between baseline and final sonoelastography was calculated for each rat.

### Detection of serum markers

Blood was drawn from each rat via the tail vein, and alanine aminotransferase (ALT), aspartate aminotransferase (AST), gamma-glutamyl transpeptidase (GGT), alkaline phosphatase (ALP) and lactic acid dehydrogenase (LDH) levels were evaluated by automated multi-channel analytical system (VITROS. 5.1 FS, Ortho-Clinical Diagnostics, Johnson & Johnson,. Raritan, NJ, USA).

### Statistical analysis

Statistical analysis was performed using GraphPad Prism software (GraphPad Software, San Diego, CA, USA). The results were presented as mean ± standard error of the mean (SE). Measurements of imaging and laboratory tests were analyzed among groups using one-one ANOVA and Mann-Whitney tests at the end of the experimental study. A p value of less than 0.05 was taken as significant.

## Results

### *Gross appearance and* histological examination of liver fibrosis induced by thioacetamide in rats

**Figure 2:**
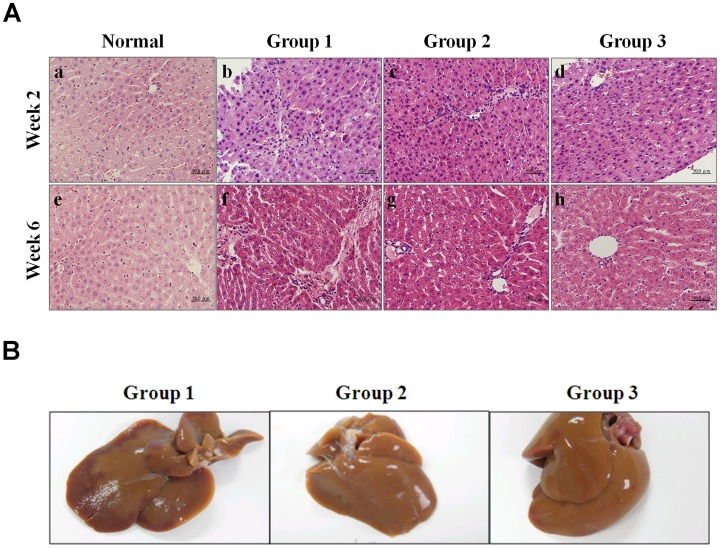
Gross appearance and histological assessments of liver fibrosis induced by thioacetamide (TAA) in rats. (A) Histological observation of liver fibrosis by hematoxylin and eosin (H&E) staining under light-field microscope with 200×magnification. The biopsies and liver tissues were sectioned in 5-µm thickness at weeks 2 (a–d) and 6 (e–f) and sections were stained with H&E. (a, e) Normal controls showed normal liver parenchyma at week 2 and week 6. (b, f) Group 1 (TAA only) showed spot necrosis of liver parenchyma at week 2 and bridging necrosis at week 6. (c, g) Group 2 (TAA +0.25 g/kg SST) showed lymphocyte infiltration at week 2 and mild fibrosis at week 6. (d, h) Group 3 (TAA+1 g/kg SST) showed relatively normal liver parenchyma at weeks 2 and 6. (B) Representative photographs at week 6 were shown. Gross examination of liver taken from Group 1 (TAA only, left panel), Group 2 (TAA +0.25 g/kg SST, middle panel) and Group 3 (TAA +1 g/kg SST, right panel) showed smooth surface.

To establish the early stage of liver fibrosis, rats were treated with TAA 2 days after administration of SST. At the end of week 2, liver tissues of rats were taken for H&E and Sirius red staining using sonography-guided core needle biopsy (Fig. 1). Small necrotic spots and bridging fibrosis were found in Group 1 (TAA only). Lymphocyte infiltration of portal area and mild periportal fibrosis were detected in Group 2 (TAA +0.25 g/kg SST). Normal tissue parenchyma and minimal periportal fibrosis were shown in Group 3 (TAA +1 g/kg SST) (Fig. 2A). At the end of week 6, the excised liver tissues from sacrificed rats were examined. The gross liver specimens showed no apparent difference in parenchyma sizes and surfaces among Groups 1–3 (Fig. 2B). H&E analysis of TAA-treated rats in Group 1 at week 6 showed damaged hepatic cells with apparent toxicity characterized by periportal hepatocytic vacoulation, centrilobular necrosis, heavy pigmentation around central veins, scattered inflammation, and giant cell transformation, compared with that in normal controls (Fig. 2A). Histological analysis also showed widely spread fibrous bands (septa), originating from portal areas and extending into the hepatic parenchyma of rats upon TAA treatment (Fig. 2A). Group 1 showed spot necrosis of liver parenchyma at week 2 and bridge necrosis at week 6. Group 2 showed lymphocyte infiltration at week 2 and mild fibrosis at week 6. Group 3 showed relatively normal liver parenchyma at week 2 and week 6. Administration of high-dose SST reduced markedly the severity of the fibrosis induced by TAA at week 6 (Fig. 2A).

**Figure 3:**
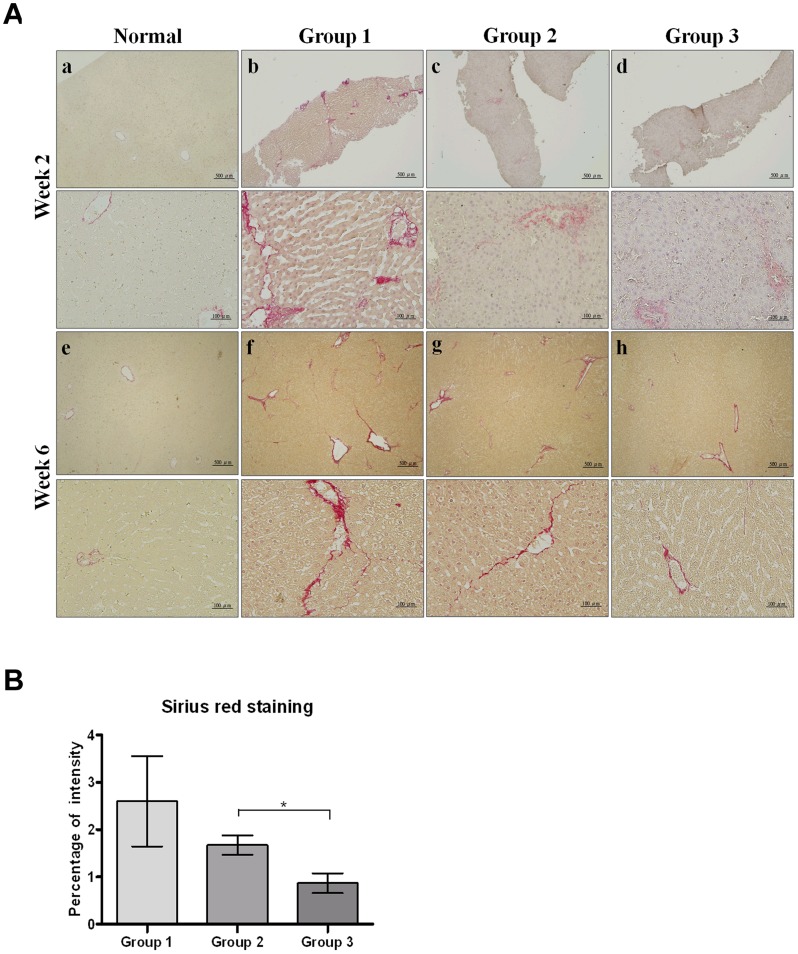
Collagen deposition in liver fibrosis induced by thioacetamide (TAA) in rats using Sirius red staining. (A) Histological observation of collagen deposition by Sirius red staining under light-field microscope with 40×(upper panel) and 200×(lower panel) magnifications. The biopsies and livers were sectioned 5-µm thickness at weeks 2 (a–d) and 6 (e–h) and sections were stained with Sirius red. (a, e) Normal controls showed no collagen deposition at weeks 2 and 6. (b, f) Group 1 (TAA only) showed extended collagen deposition and large septa of hepatic lobules, (c, g) Group 2 (TAA +0.25 g/kg SST) reduced the severity of hepatic fibrosis at weeks 2 and 6. (d, h) Group 3 (TAA +1 g/kg SST) markedly reduced the severity of hepatic fibrosis at weeks 2 and 6. (B) Quantitative analysis of Sirius red staining of each group at week 6. In comparison with Group 1, Groups 2 and 3 show decreased percentages of immune intensity. Bar, SE; *p<0.05.

Extended collagen deposition and large septa of hepatic lobules were observed by Sirius red staining in Group 1 compared with normal controls. Compared to Group 1, extended collagen deposition was decreased in Groups 2 and 3 (Fig. 3A). After quantification, the percentages of fibrosis intensity of the three groups were as follows: 2.6±0.95 (Group 1, TAA only), 1.67±0.2 (Group 2, TAA +0.25 g/kg SST) and 0.86±0.21 (Group 3, TAA +1 g/kg SST) (Fig. 3B). The fibrosis stained by Sirius red in Group 1 was higher than that in Groups 2 and 3, indicating staining in groups with pretreatment of low (Group 2) or high (Group 1) dose of SST. According to the Ishak histological scores, the median of liver fibrosis at week 6 were 3, 2 and 1 in Groups 1, 2 and 3, respectively. In addition, comparison of the Metavir liver fibrosis assessment scale also showed that high-dose SST administration decreased the extent of liver fibrosis induced by TAA from F2 down to F1 (Table 1). By administration of SST, our animal model of TAA-induced liver fibrosis has successfully developed the different stages of early liver fibrosis.

**Table 1:** Evaluation of TAA-induced liver injury in rat models using Ishak and Metavir scores.

	Ishak score	Metavir score
Group 1	3	2
Group 2	2	2
Group 3	1	1

Group 1 (TAA only); Group 2 (TAA+0.25 g/kg SST); Group 3 (TAA+1 g/kg SST).

TAA: Thioacetamide; STT: Sho-saiko-to; Fibrosis score: Ishak: 0–6; Metavir: F0 = 0, F1 = 1, F2 = 2, F3 = 3. Data represent as median.

### Detection of liver fibrosis induced by thioacetamide in rats using gadoxetic acid-enhanced MRI

**Figure 4:**
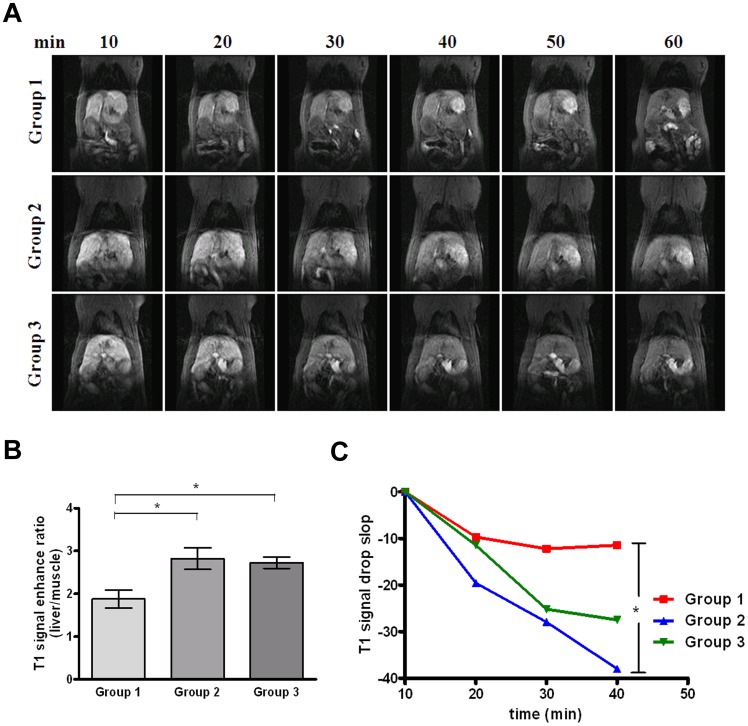
Examination of thioacetamide (TAA)-induced liver fibrosis in rats by gadoxetic acid-enhanced MRI. (A) T1-weighted MRI images of Group 1 (TAA only, upper panel), 2 (TAA +0.25 g/kg SST) and 3 (TAA +1 g/kg SST) were shown at different time points (10, 20, 30, 40 50 and 60 min). (B) Quantification of ratios of relative enhancement of each group at time point of 10 min. Significant decreases in ratios were observed among Groups 1, 2 and 3. Bar, SE; *p<0.05. (C) Quantification of the signal drop slope in each group was determined by dividing the ratio difference at different points by the ratio at 10 min. A significant drop of the slope was observed between Groups 1 and 2. *p<0.05.

Typical T1-weighted images of liver at 10–60 min after gadoxetic acid injection in each group are shown in Fig. 4A. At the time point of 10 min, the ratio of relative enhancement in Group 1 (TAA only) was significantly smaller than that of the other groups. The ratios of the three groups were as follows: 1.87±0.21 (Group 1, TAA only), 2.82±0.25 (Group 2, TAA +0.25 g/kg SST) and 2.72±0.12 (Group 3, TAA +1 g/kg SST) (Fig. 4B). The decrease in ratio was more significant in Group 1 than in Groups 2 and 3 (p<0.05). Further examining the slope of signal drop reveals that the slope in Group 1 was not as steep as that in Groups 2 and 3 (p<0.05) (Fig. 4C). Compared with Group 1, Groups 2 and 3 showed sharp T1 signal drop slopes (Fig. 4C). According to these two measurements of gadoxetic acid-enhanced MRI, there was no significant difference between Groups 2 and 3, indicating that it is not possible to differentiate the subtle progression or regression of early liver fibrosis using this device.

**Figure 5:**
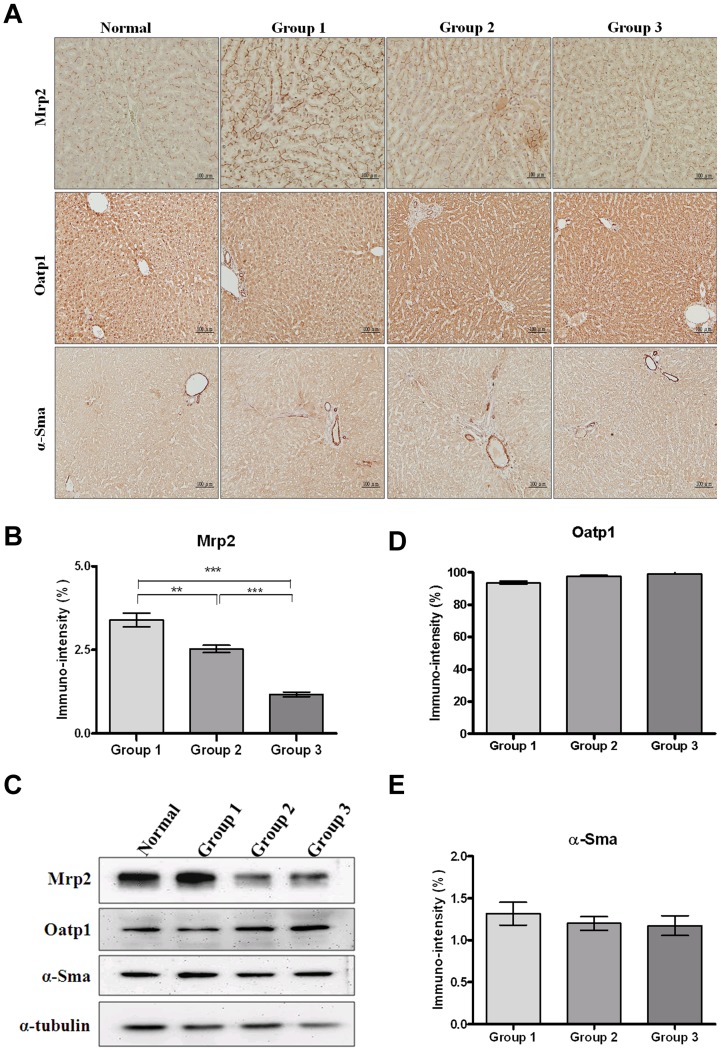
Detection of Mrp2, Oatp1 and α-Sma expression in liver fibrosis induced by thioacetamide (TAA) in rats by immunohistochemistry and Western blots. (A) Immunohistochemistry of Mrp2 (upper panel), Oatp1 (middle panel) and α-Sma (lower panel) in rat liver sections of normal controls, Group 1 (TAA only), 2 (TAA+0.25 g/kg SST) and 3 (TAA+1 g/kg SST) was shown under light-field microscope with 200×magnifications. (B) Graph showed quantification of percentage of positive Mrp2 in Groups 1, 2 and 3. Bar, SE; **p<0.01; ***p<0.001. (C) Western blots of liver tissues from Mrp2, Oatp1 and α-Sma in rat liver tissues of normal controls, Groups 1, 2 and 3. α-tubulin serves as internal control. (D) Graph showed quantification of percentage of positive Oatp1 in Groups 1, 2 and 3. Bar, SE. (E) Graph showed quantification of percentage of positive α-Sma in Groups 1, 2 and 3. Bar, SE.

In view of the organic anion-transporting polypeptide-1 (Oatp1) and multidrug resistance protein 2 (Mrp2) dependent on intracellular uptake of gadoxetic acid in the liver [18], [19], the expression of Mrp2 and Oatp1 proteins were examined by IHC and Western blot. The immune intensity of Mrp2 expression relative to normal controls was as follows: 3.39±0.2 (Group 1, TAA only), 2.52±0.11 (Group 2, TAA +0.25 g/kg SST) and 1.17±0.06 (Group 3, TAA +1 g/kg SST). Relative to that of normal controls, the Mrp2 expression in Group 1 was significantly higher than that in the TAA-treated groups (Figs. 5A and 5B). Similar result was shown in Western blot (Fig. 5C). After IHC quantification by measuring the immune intensity, the Oatp1 levels of the three groups were as follows: 93.84±1.04 (Group 1, TAA only), 97.8±0.63 (Group 2, TAA +0.25 g/kg SST) and 99.18±1.3 (Group 3, TAA +1 g/kg SST). There was no significant difference in Oatp1 expression among the groups (Figs. 5A and 5D), indicating that hypofunction of Mrp2 (rather than Oatp1) might be related to the transport of gadoxetic acid in TAA-induced fibrotic livers.

### Detection of liver fibrosis induced by thioacetamide in rats using sonoelastography

**Figure 6:**
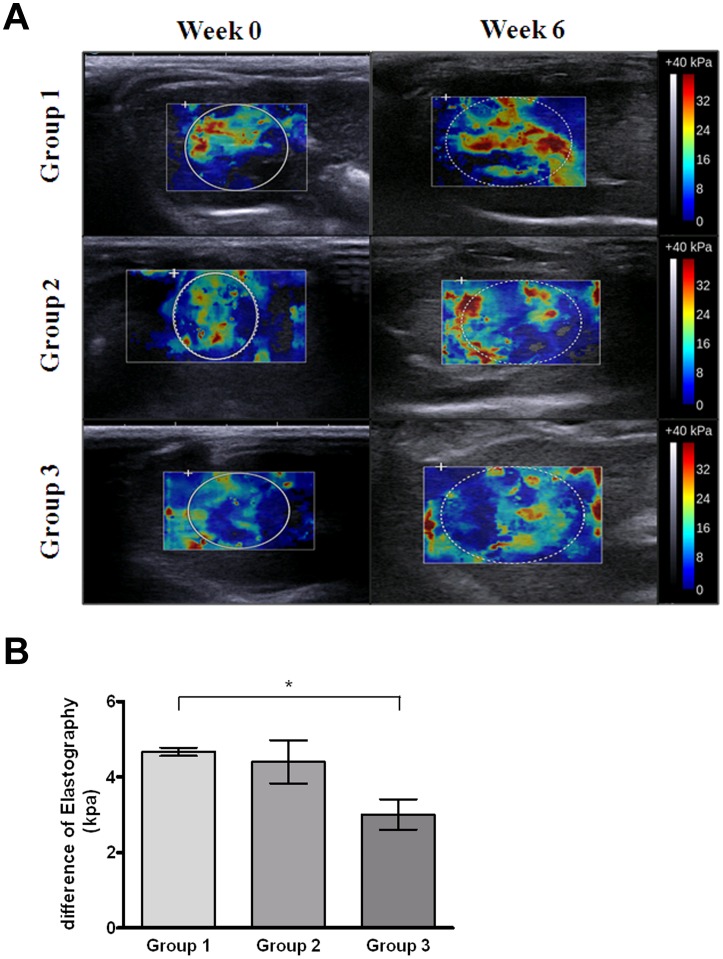
Examination of thioacetamide (TAA)-induced liver fibrosis in rats by sonoelastography. (A) Sonoelastographic images before and after SST administration (weeks 0 and 6) in Groups 1 (TAA only, upper panel), 2 (TAA +0.25 g/kg SST, middle panel) and 3 (TAA +1 g/kg SST, lower panel). In the elastography frame, blue color stands for hard issues and red stands for soft issues. (B) Quantification of difference in liver stiffness of each group at weeks 0 and 6 by sonoelastography. Significant decrease in liver stiffness was observed in Groups 1 and 3. Bar, SE; *p<0.1.

Sonoelastography has recently been introduced as a new and useful method for non-invasive assessment of liver fibrosis [20]. To evaluate the possibility for detection of fibrosis induced by TAA in rats, sonoelastography was performed before (week 0) and after induction of TAA (week 6). Group 1 (TAA only) had more red-colored signals representing hard tissues than blue-colored ones for soft tissues (Fig. 6A). After quantitative measurement, the mean difference before and after experiments in Groups 1 (TAA only), 2 (TAA +0.25 g/kg SST) and 3 (TAA +1 g/kg SST) were 4.66±0.1, 4.4±0.57 and 3±0.4 KPa, respectively (Fig. 6B). The measurements of liver stiffness were significantly higher in Group 1 than in Group 3 (p<0.1), while the liver stiffness was higher in Group 2 than in Group 3. There was a decreased trend among Groups 1, 2 and 3 even thought there was no significant difference between Groups 1 and 2.

In view of the association between α-smooth muscle actin (α-Sma) with stiffness in the liver [21], the expression of α-Sma was examined by IHC and Western blot (Figs. 5A and 5C). Immunohistochemical analysis results show that α-Sma expression was detected mostly in walls of the vascular structure in each group (Fig. 5A). After quantification of IHC by measuring the immune intensity, the α-Sma expression of three groups were as follows: 1.31±0.13 (Group 1, TAA only), 1.2±0.08 (Group 2, TAA +0.25 g/kg SST) and 1.17±0.11 (Group 3, TAA +1 g/kg SST). Although there was no statistically significant difference in α-Sma expression, a decreased trend of α-SMA expression was observed in these three groups (Fig. 5E). Western blots also show similar results (Fig. 5C).

### Comparison of liver function in liver fibrosis induced by thioacetamide in rats by serum proteins

Liver damage is divided into hepatocellular damage with elevated AST and ALT; and cholestatic damage with an increase ALP and GGT [22]. To evaluate if the well-established serum proteins show differentiated capability in mild liver fibrosis, serum levels of liver function enzymes, including AST, ALT, GGT, ALP and LDH were employed to monitor hepatic injury after TAA treatment and recovery by SST administration. As shown in Table 2, there was no significant difference in serum levels of AST, ALT, GGT and ALP between the three experimental groups and normal rats. Only the LDH levels in the three experimental groups were higher than those of normal rats even though the difference was not statistically significant. However, the LDH levels of the three experimental groups could not be differentiated in a stage-dependent manner, indicating that LDH was not a promising biomarker in detection of chemopreventive effects on administration of SST in TAA-induced liver fibrosis (Table 2).

**Table 2:** Comparison of biochemical markers in TAA-induced liver fibrosis in rats.

Group	AST (U/L)	ALT (U/L)	GGT (U/L)	ALP U/L)	LDH (U/L)
Normal	28±6	49±4	<12	<14	1522±404
Group1	33±7	54±2	<12	<14	3071±1346
Group 2	44±9	54±5	<12	<14	3907±1522
Group 3	33±9	46±1	<12	<14	3310±1414

Group 1 (TAA only); Group 2 (TAA+0.25 g/kg SST); Group 3 (TAA+1 g/kg SST).

TAA: Thioacetamide; STT: Sho-saiko-to; AST: Alanine aminotranferease; ALT: Aspartate aminotransferase; GGT: Gamma-glutamyl transpeptidase; ALP:Alkaline phosphatase; LDH: Lactic acid dehydrogenase. Data represent as mean±SE.

## Discussion

The administration of TAA in rats is a commonly used model of chemically induced liver cirrhosis, and the cirrhosis induced by TAA is supported to be morphologically well defined and uniform, reflecting the major features of human diseases [23] [23]. In contrast to other studies using TAA-induced liver fibrosis in rat models, this research used intermittent intraperitoneal injection of TAA twice weekly for 6 weeks instead of 12 weeks (Fig. 1) [24]. Our method can achieve moderate hepatic fibrosis without additional acute side effects. The liver tissue surface, H&E staining as well as biochemistry tests did not show any apparent change among the experimental animals (Fig. 2 and Table 2). Using Sirius red staining for collagen detection, the accumulation of collagen fiber in the three experimental groups showed a declined trend (Fig. 3). The Ishak score of the group administered with TAA can reach only 3 while that of the group receiving high-dose SST treatment downgraded to 1 (Table 1). This study showed that the modified animal model is suitable for studying relatively early liver fibrosis and evaluating the effects of anti-fibrotic therapy.

There are several biological effects showing that SST improves liver function. The mechanisms of SST are probably related to anti-inflammatory, regulation of matrix metalloproteinase and natural killer cell, immune-modulating effects and reducing the effects of toxic drugs [4], [5]. While anti-liver fibrosis and cirrhosis therapeutic effects were obtained in animal experiments and clinical use on certain results, its mechanism of action against liver fibrosis has not been fully understood. In this study, administration of SST indeed reduced TAA-induced accumulation of collagen fibers (Fig. 3), increased the ratio of T1 relative enhancement as seen in gadoxetic acid-enhanced MRI, and reduced liver stiffness as observed by sonoelastography (Figs. 4 and 6).

Performing a liver biopsy serves three purposes: (1) for diagnosis, (2) for assessment of prognosis (disease staging), and/or (3) for assistance in making decisions on therapeutic management. Although liver biopsy is considered a safe procedure when performed by experienced operators, the mortality rate is less than or equal to 1 in 10,000 liver biopsies [25]. Additionally, liver biopsy is difficult for monitoring the interval changes of liver fibrosis. Non-invasive tools, including circulating biochemical markers and imaging devices, may be considered to replace the use of liver biopsy in assessing the progression of liver fibrosis [26].

Elevated levels of serum proteins, such as AST, ALT ALP and GGT, were frequently examined in liver damage [22]. There was no apparent difference in levels of AST, ALT, ALP or GGT among healthy rats and Groups 1–3 experimental rats (Table 2). Laboratory data in our animal study indicated only minimal hepatocellular and cholestatic damage, but evident liver fibrosis using our TAA protocol. AST, ALT and LDH can be related to staging of liver fibrosis in human and rat studies [27], [28]. Our study only demonstrated increased serum LDH levels in experimental groups in comparison with normal controls indicating that imaging of gadoxetic acid-enhanced MRI and sonoelastography can detect minimal liver fibrosis more than biochemistry tests did.

Gadoxetic acid is highly liver-specific with an uptake of about 50% into hepatocytes of injected dose. Owing to its accumulation in the hepatocytes, a pronounced enhancement and a signal increase of normal liver tissue is seen. It would be assumed that gadoxetic acid uptake in the liver would decrease in the setting of hepatic fibrosis as more gadoxetic acid is excreted through renal pathways rather than via hepatocytes, but gadoxetic acid deposition is more likely to be dependent on the balance between the hepatocyte uptake and biliary excretion. OATP1, which transports gadoxetate disodium into the hepatocytes, has decreased uptake levels in cirrhotic rats and humans [29]. Tsuda et al. reported that signal enhancement in the TAA-treated group was significantly lower than that in the control group using gadoxetic acid-enhanced MRI [19]. Similar to our results, gadoxetic acid-enhanced MRI demonstrated higher ratio of T1 signal enhancement detected in mild fibrotic liver (Ishak scores 1–2) than in severe fibrotic liver (Ishak score 3). In comparison with blood laboratory tests, gadoxetic acid-enhanced MRI is sensitive enough to detect changes in fibrosis in relation to response to anti-fibrotic therapy (Fig. 6 and Table 2). It is well known that Oatp1 regulates the uptake of gadoxetic acid by hepatocytes and that Mrp2 mediates the biliary excretion of gadoxetic acid [18], [30], [31]. It has been reported that the expression of Oatp1 and Mrp2 decreased in cases of hepatitis or cirrhosis [32], [33]. It is thought that the down-regulation of Oatp1 induces the reduction of gadoxetic acid uptake by hepatocytes, and the down-regulation of Mrp2 leads to the accumulation of gadoxetic acid in hepatocytes. In contrast, Tsuda et al. and our study demonstrated that the elevated expression of Mrp2 would lead to a significant signal intensity decrease on gadoxetic acid-enhanced MRI in TAA-treated liver fibrosis [34]. Our findings may indicate that the reduction of TAA-induced fibrosis by SST treatment in rats appeared to be accompanied with impaired function of Mrp2 proteins. However, the impact of the experiment Mrp2 transporter protein, without affecting the performance of the mechanism Oatp1 transporter protein, still merits further analysis of the compound effects. Given that SST did not effectively reduce plasma ALT, AST and other circulating enzymes (Table 2), whether the experimental results obtained by this animal model induced by mild fibrosis is related to SST in the experimental model and regulation under the involvement of the immune or inflammatory response has to be further confirmed.

Ultrasound-based elastography has been extensively evaluated as a non-invasive tool to assess liver fibrosis; and measurement of liver stiffness has high sensitivity and specificity for detecting histological cirrhosis of liver [35]. Our results showed that elasticity measured *in vivo* with sonoelastography was less stiff after 6 weeks in rats administered with high- and low-dose SST in a TAA-induced liver fibrosis model. Our study suggested that sonoelastography may be useful in monitoring the therapeutic effect of preventing liver fibrosis and demonstrated that sonoelastography is capable of distinguishing the histological changes in early fibrosis over time in relation to response to anti-fibrotic therapy in comparison with blood laboratory tests (Fig. 6 and Table 2). There is a good correlation between liver stiffness measured with sonoelastography and cirrhosis of liver. Patients with elevated serum ALT levels but the same degree of liver fibrosis had higher liver stiffness measurement values [36]. According to the minor histological liver injury in our study models, the possibility for inadequate measurement in the blood laboratory tests can be excluded. The efficacy of gadoxetic acid-enhanced MRI and sonoelastography for monitoring liver fibrosis was compared and sonoelastography has shown better performance in detection of subtle changes in mild liver fibrosis (Figs. 4 and 6). Clinical applications of imaging tests for monitoring early liver fibrosis need to be validated by future human study and different experimental animal models.

## Conclusion

Sonoelastography and gadoxetic acid-enhanced MRI could differentiate the early fibrotic livers developed by administration of SST in rats with TAA-induced liver fibrosis when compared with the laboratory tests.
